# EARLY-LIFE SOCIOECONOMIC POSITION AND ACCUMULATION OF HEALTH-RELATED DEFICITS IN MIDLIFE

**DOI:** 10.1093/geroni/igz038.831

**Published:** 2019-11-08

**Authors:** Nina Rogers, Chris Power, Snehal P Pereira

**Affiliations:** 1 University College London, London, United Kingdom; 2 University College London, London, England, United Kingdom

## Abstract

Improved understanding of predictors of frailty is key to delaying its onset. Yet, few studies have examined whether early-life socioeconomic position (SEP) predicts frailty in midlife. In the 1958 British Birth Cohort (n=7601), we examined (i) associations between early-life SEP and frailty at 50y and (ii) whether associations were due to continuities in disadvantage into mid-adulthood. Frailty was measured using an index composed of 37 health-deficits. Associations between early-life SEP and frailty were examined using linear regression. Lower early-life SEP was associated with higher frailty, e.g. compared to professional/managerial class, the frailty index was higher by 3.09% (95% CI:-0.65%, 6.84%) for skilled non-manual, 10.8% (8.20%, 13.4%) for skilled manual and 14.2% (11.1%, 17.2%) for partly skilled/unskilled. After adjustment for adult disadvantage, the trend remained, albeit weaker. Findings suggest that interventions in mid-adulthood targeted to those exposed to early-life disadvantage could reduce the risk of developing frailty when entering later life.

